# The Breast Edema Enigma: Features, Diagnosis, Treatment, and Recommendations

**DOI:** 10.7759/cureus.23797

**Published:** 2022-04-03

**Authors:** Sarina S Gupta, Harvey N Mayrovitz

**Affiliations:** 1 Osteopathic Medicine, Nova Southeastern University Dr. Kiran C. Patel College of Osteopathic Medicine, Fort Lauderdale, USA; 2 Medical Education, Nova Southeastern University Dr. Kiran C. Patel College of Allopathic Medicine, Davie, USA

**Keywords:** tissue dielectric constant, guidelines, diagnostic recommendations, breast-conserving therapy, breast cancer, breast lymphedema, breast edema

## Abstract

Breast edema most commonly occurs after breast cancer treatment involving breast-conserving therapy, although it may have a variety of other causes. As compared to research on breast cancer treatment-related lymphedema, breast edema and its objective measurement and diagnosis is far behind. Consequences of this disparity contribute to uncertainty and variability in its diagnosis, its treatment, and even the characterization of its incidence and morbidity. Moreover, consensus on a standardized definition, objective diagnostic method, and treatment techniques for breast edema has not yet been reached, making it difficult to provide appropriate guidelines with respect to its management. Given the recent rise in breast edema incidence as an outcome of the increasing use of breast-conserving therapy, this timely review examines the current state of breast edema assessment and makes a case for standardization in part via quantitative methods to diagnose and track breast edema.

## Introduction and background

Breast edema is fluid accumulation within breast tissue that may lead to visible swelling, increased breast parenchymal density, inflammation, skin dimpling (peau d’orange) and thickening, breast pain, and sagging of the breast [1]. This fluid buildup is largely a result of compromised outflow due to an obstruction or disruption of lymphatic vessels that drain the breast, which may occur from a diverse spectrum of etiologies. The predominant cause is breast cancer treatment referred to as breast-conserving therapy (BCT) whereby breast-conserving surgery (also called lumpectomy or segmental mastectomy) is performed, followed by radiotherapy [2-4]. Often coexistent with breast edema attributable to BCT is lymphedema of the arms, an entity that has been far more recognized and treated clinically as well as extensively explored and discussed in the literature [1,2]. Because breast edema is often a painful condition and because of the psychological, social, personal, physical, and professional repercussions associated with BCT-induced breast edema, it is important for clinicians and researchers to be familiar with this condition. Moreover, as minimally invasive management of breast cancer is becoming increasingly common, the percentage of persons so treated and who have subsequent breast edema was reported as 24.8%, with prevalence peaking at six months post-radiotherapy [2]. Management of breast edema can be facilitated if both the patient and the clinician are aware of its presentation, its course, and methods used for its detection and monitoring. If left untreated, breast edema can have psychological effects leading to decreased quality of life, body image issues, and fear of cancer reoccurrence, as well as continued breast discomfort and pain [2,5].

Besides BCT, other less common causes of breast edema include inflammatory breast carcinoma, congestive heart failure, mastitis, lymphatic obstruction (by axillary, chest wall, or intrathoracic lesions), metastasis, breast lymphoma, and trauma [1,6]. Furthermore, bilateral edema and unilateral breast edema often connote distinct etiologies and methods of management [6,7]. The onus is thus on the physician to obtain a detailed history, physical examination, and appropriate testing in order to avoid a misdiagnosis and to provide an effective, personalized treatment regimen.

Current methods to detect breast edema incidence tend to be suboptimal, often limited to subjective assessments (such as surveys, questionnaires, and self-reports) and clinical assessments based on cursory physical examinations [8]. This is in part due to the absence of easily applied objective measures. These factors coupled with the often enigmatic presentation of breast edema in the absence of discernible swelling can make it difficult to formulate a proper breast edema diagnosis and may explain why the reported incidence of breast cancer treatment-related breast edema has a wide range (10% to 90.4%) [2,9]. A standardized diagnostic methodology that reliably and reproducibly detects and tracks breast edema over time in a clinically friendly way would be a welcomed advance as is subsequently discussed.

## Review

The objective of this paper is to bring to the forefront the clinical entity of breast edema through a thorough literature search, highlighting the need for a standardized definition and diagnostic methodology. We suggest the use of tissue dielectric constant (TDC) values as a tool to establish quantitative parameters to assess the presence and progression of breast edema, subject to corroboration by further research.

Search strategy

Five databases (PubMed, CIANHL Complete, Web of Science, Embase, and Biomedical Reference Collection: Comprehensive) were searched for papers. Each search was performed by using the words “breast” and “edema” as two separate terms, both under the “title” search category. An initial combined total of 408 records were produced. Duplicate records and those that were not peer-reviewed were eliminated. Abstracts of the 70 remaining non-duplicate returns were read. Papers focused primarily on lymphedema outside the breast were subsequently eliminated as well. Other relevant articles (N=29) that did not appear in the original database search, because they did not have the words “breast” and “edema” in their titles, were also considered for inclusion. All articles were found because they had been cited in papers that did appear in the previous database searches. Although these 29 articles do not specifically address breast edema, they were still considered for inclusion because they discuss related topics that are relevant to this paper, including TDC, breast cancer, and magnetic resonance (MR) mammography. Our inclusion criterion thus established that articles must be peer-reviewed, and must explicitly state and discuss at least one of the following to be eligible: breast edema (of any etiology), TDC, MR mammography, or breast cancer.

From the 79 articles that met inclusion criteria, a total of 53 (N=53) were referenced in our paper since all case studies were eliminated. One exception was made for a case report that provided novel and relevant information regarding the use of MR mammography in breast edema diagnosis.

**Figure 1:**
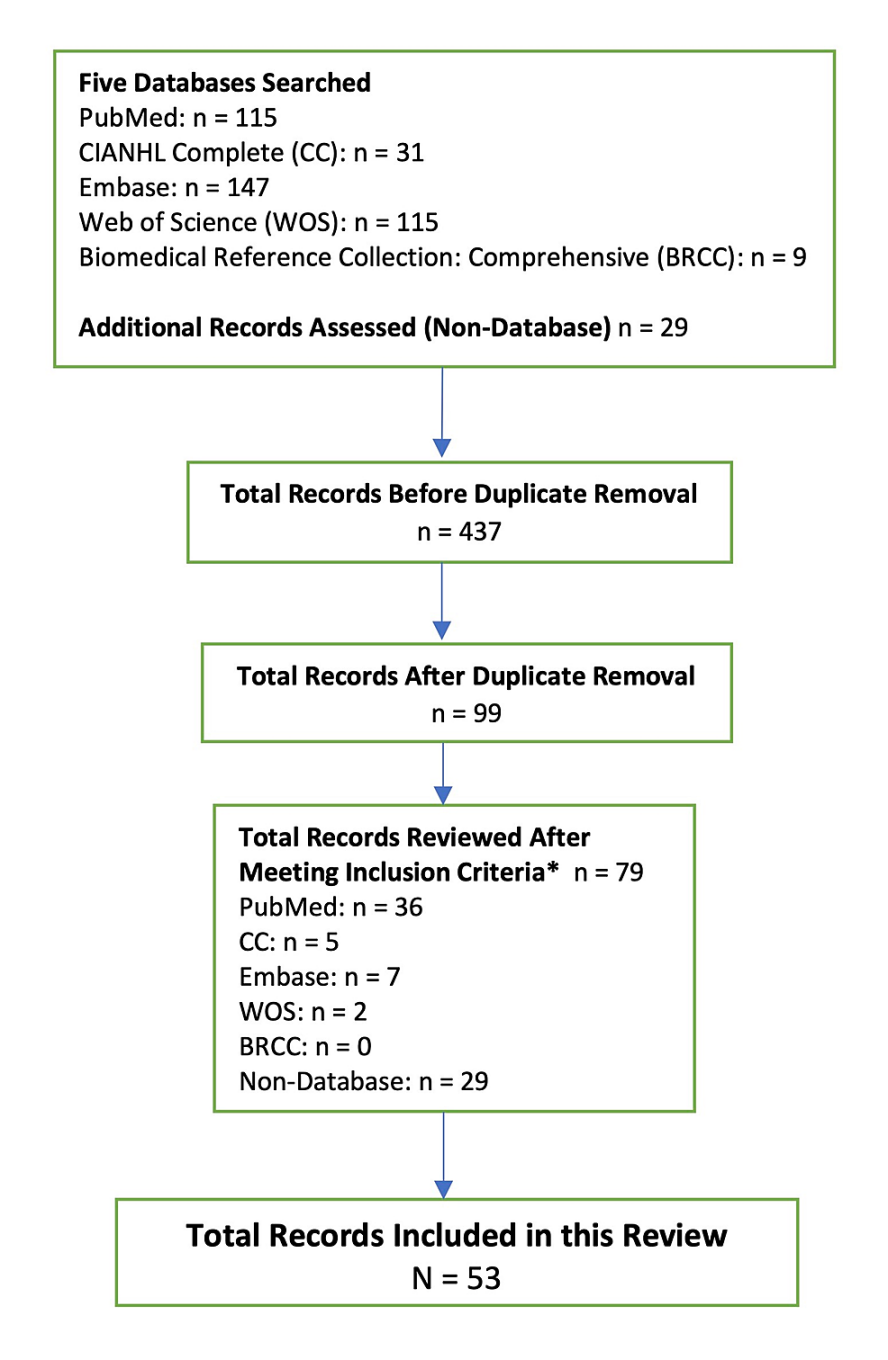
Search strategy and outcomes The inclusion criteria (*) was that the article must be peer-reviewed and must explicitly state and discuss at least one of the following: breast edema (of any etiology), tissue dielectric constant, MR mammography, or breast cancer. MR, magnetic resonance

The final list of references spanned a wide date range, from 1959 to 2021. A flowchart to delineate the article search, review, and elimination process is shown in Figure 1. Prior to submission, the five databases used were revisited to account for any additional publications on breast edema that had been added after the initial search, and none were found.

Diagnosis and management of breast edema

There are multiple approaches that have been put forward to diagnose and manage breast cancer that include macroscopic diagnosis approaches, imaging approaches, and objective measurement approaches.

Macroscopic diagnostic features

Some key features of breast edema include a characteristic “peau d’orange” sign (dimpling of the skin that resembles an orange peel), heaviness of the breast, skin thickening, erythema, hyperpigmented skin pores, and pitting edema [1,10]. Breast hardening is also a side effect of radiotherapy treatment in breast cancer patients because irradiation produces hardening of fat, and the breast tends to have a high content of adipose tissue. Researchers have posited different methods to categorize the varying manifestations of breast edema, from parenchymal breast edema or “generalized enlargement or swelling of breast tissue itself” to cutaneous breast edema or “changes in epidermis and dermis” [11] and to the stage 1 to 3 classifications as dictated by changes in breast volume, appearance, and perceived pain [12].

Breast imaging techniques

To visualize breast edema, several non-invasive imaging techniques have been employed. X-ray findings detecting breast edema date back to at least 1959 in the literature [13]. Mammograms and ultrasound imaging are useful diagnostic tools in assessing increased parenchymal density, a characteristic feature of breast edema. Ultrasound has also been used to detect skin thickening characteristic of edema that is visualized as a hyperechoic region [14]. Contrast-enhanced digital mammography (CEDM) may supplant plain radiography and ultrasonography since the beams from these two procedures may be attenuated by the presence of dense tissue and also fluid associated with breast edema [15]. Moreover, CEDM is cost-effective and can produce high-resolution, contrast-enhanced, low-energy images.

Currently, magnetic resonance imaging (MRI), with its high sensitivity, plays a crucial role in diagnosing breast cancer and characterizing tumor stage, morphology, margins, and neoangiogenesis [16], particularly when performed with contrast enhancement [17]. Its use to evaluate breast edema is optimized by T2 diffusion-weighted imaging in which thermal movement of water molecules in extracellular fluid is detected as a high-intensity signal [18,19]. The edema may be focal (including subcutaneous and peri-tumoral subtypes) or diffuse. Numerous studies have confirmed that focal edema is associated with malignancy in most cases [16,20-22]. MR mammography has also been studied and utilized extensively to analyze and demonstrate a correlation between breast edema and various features of tumor aggressiveness [22-25]. For example, it was found that peri-tumoral edema surrounding the tumor that was detected by T2-weighted MRI was significantly associated with cancer cell invasion of lymphatics, blood vessels, and other histopathological changes, with p-values < 0.001 [26]. MRI detection of edema surrounding the tumor may therefore predict poor prognosis in breast cancer patients with this type of breast edema; moreover, it has also been reported that the amelioration of this edema after chemotherapy, based on subsequent MRI findings, may serve as a favorable prognostic indicator [27].

Tissue dielectric constant measurements

Despite these previously described classifications and diagnostic techniques, quantitative measurement tools have been extremely limited for breast edema. Recent work has indicated that localized measurements of breast tissue water via TDC measures may be a useful objective measure [28]. TDC measurements in any area of the body work by placing an open-ended coaxial probe in contact with the skin whereby a low-level 300-MHz electromagnetic wave is delivered to the tissue [29]. The wave is partially absorbed by the tissue, and the remainder is reflected and processed by a device to determine TDC. The size and phase of this electromagnetic wave differ based on the dielectric constant, which is indicative of the relative permittivity of skin-to-fat tissue. At 300 MHz, the dielectric constant predominantly depends on polarization of water molecules and is commensurate with total tissue water content, including both extracellular and intracellular water molecules [30]. In general, at any given frequency above 100 MHz, probes larger in diameter penetrate more deeply into the skin, and the measured dielectric is thus a product of the dielectric properties of multiple layers of skin and of subcutaneous fat [31].

While TDC measurements for breast edema initially assessed absolute TDC values at a single timepoint [32] and were thus generally unable to detect the earlier stages of breast edema, a recent study demonstrates the efficiency and potential of utilizing absolute TDC values as a method to assess changes in breast edema over time [28]. Furthermore, this study is the first to establish an evidence-based mathematical calculation yielding an absolute threshold value of 41, attained by comparison of TDC values from healthy versus pre-biopsy tumorous breasts. Comparing these TDC values simultaneously allowed these researchers to obtain a standard site inter-breast TDC threshold ratio of 1.28. This inter-breast ratio is comparable to the post-surgical ratio of 1.4 reported by other pioneers in the field [32,33]. Having these baseline values prior to intervention can serve as powerful tools for early detection as well as tracking treatment progress and success. The diagnostic value of TDC in assessing breast cancer-related lymphedema extends beyond the breast as well, as it has been used to analyze lymphedema of the arms, hands, and trunk, in addition to non-breast cancer-related lower limb lymphedema [28,29,34-40].

TDC has also been used in conjunction with qualitative diagnostic methodologies to conduct a thorough appraisal of breast edema. A recent study undertook this assessment by utilizing TDC in addition to indocyanine green (ICG) lymphography, an emerging imaging technique for qualitative detection of breast edema and visualization of breast lymphatics [41]. Their study included 20 participants, of whom 10 were healthy non-edematous controls and 10 had previously undergone breast cancer surgery. ICG lymphography showed a high sensitivity in detecting morphologic changes in lymphatic flow in 100% of breast cancer patients compared with the 0% seen in the healthy controls. Moreover, quantitative measurements using TDC in these same participants showed a sensitivity of 60% for those with breast edema when whole breast measurements were performed. This finding supports neoteric evidence emphasizing the importance of assessing one breast quadrant utilizing TDC measurements over whole breast measurements in order to obtain accurate and reproducible findings with higher sensitivity.

Management of breast edema

As a final note, research on the efficacy of current breast edema management options and, by the same token, on the advancement of breast edema treatment overall is paltry. One of the main treatment techniques utilized for edema following breast cancer surgery is complex decongestive therapy [42]. This treatment decreases interstitial fluid by redirecting excess lymph back into the body’s main circulation system by means of manual lymphatic drainage (MLD), compression banding, lymph-reducing exercises, and skin care [41]. The latest systematic review, however, published in 2021, recommends temporary and potentially permanent removal of MLD due to lack of research, insufficient proof of effectiveness [43], high cost, and its time-consuming nature [1]. Another proposed intervention technique is kinesiology taping, wherein medical tape is applied to pull the breast skin off the underlying pectoralis muscle fascia to improve lymphatic drainage. This, in turn, decreases swelling and pain, and enhances muscle activity [44,45]. Recently, the use of compression vests and sports bras to reduce symptoms of breast and chest wall edema has also been considered and evaluated [33,46]. Evidence proving the efficacy of these treatments, nonetheless, is lacking in the literature. There exists no clear directive on the optimal management of breast edema. It may, therefore, be of paramount importance to focus on early detection, heightened recognition and awareness of the condition by both the physician and the patient, and patient education on the typical clinical course of breast edema and its available treatment options in order to avert the more serious aftermath of breast edema [5].

Importance of early and accurate detection of breast edema

Breast edema has become an increasingly research-worthy and relevant topic as its incidence has increased hand-in-hand with the rise of BCT, attributable to technological advances in earlier detection and minimally invasive techniques. A common form of BCT includes breast-conserving surgery followed by radiotherapy. While BCT is the current standard of care for breast cancer in its initial stages and offers multiple benefits including safety, efficiency, and treating the cancer itself while preserving the breast, a possible side effect is isolated lymphedema in the salvaged breast [47]. Given the state-of-the-art nature of BCT, the first significant insight into the effects of two widely used BCT techniques, oncoplastic surgery and neoadjuvant chemotherapy, on breast edema was published in 2019 by Young-Afat et al [2]. Their analysis of these methodologies in conjunction with other previously assessed forms of BCT allowed them to ascertain multiple risk factors for breast edema resulting from BCT. These risk factors include axillary lymph node drainage (which was associated with breast edema at all timepoints), oncoplastic surgery, a larger tumor size, locoregional radiotherapy, local radiotherapy boost, and adjuvant chemotherapy. Furthermore, their study included a systematic analysis of the impact of breast edema on health-related quality of life as reported by patients themselves - a topic that has been, and continues to remain, largely neglected in breast edema research thus far. Importantly, they found a significant association between breast edema and increased breast pain throughout the entire course of their study, which assessed breast edema prior to initiation of radiation treatment and at 3, 6, 12, and 18 months after treatment, as well as significant associations at baseline and six months between breast edema, and impaired quality of life, physical functioning, and body image [2]. Their findings are not only consistent with the conclusions drawn from previous research, but their ability to obtain concrete, scientific, quantitative data also help fortify the results of these past findings on breast edema. They highlight the vital need for early recognition of breast edema, for targeted interventions, and for research analyzing these interventions in order to assess efficacy in terms of treating breast edema.

Early detection of breast edema is crucial for numerous reasons. It allows for prompt treatment and adequate control of disease, preventing progression to fulminant disease. It also enables physical alleviation of present symptoms, possibly precluding psychological consequences that may result from impaired quality of life from progressive disease. Afflicted patients often express symptoms such as breast pain, which make it difficult for them to perform various tasks such as comfortably wearing a fitted bra [44,48] and sleeping peacefully through the night [49]. Patients also report an impaired quality of life due to fear of injury, recurrence of cancer, poor outlook for the future, anxiety, embarrassment, relationship issues, and family issues (family members are unsure of how to help mitigate their symptoms) [5,50,51]. Moreover, these psychosocial stressors can go unchecked and progress if the clinician does not address them. This underscores the importance of physicians diagnosing breast edema and acknowledging the gamut of physical, psychological, and social issues that accompany this condition. Only then can a physician understand the patient’s perspectives and concerns in a compassionate manner and co-construct a personalized treatment plan that can target all facets of breast edema [44].

Equally as important as early diagnosis is accurate diagnosis of the source of breast edema to render the most appropriate treatment, especially given the multitude of etiologies that exist. This concept has been expressed in the literature for many years. In 1987, Seibel et al. published the first mammography-proven report of a patient with unilateral edema due to an obstructed superior vena cava that mimicked inflammatory carcinoma [52]. His case report thus accentuated the importance of ruling out inflammatory carcinoma in patients with unilateral breast edema presenting with skin thickening and subcutaneous changes. More recently, Todd et al.’s 2017 publication noted that “misdiagnosis of breast oedema with inflammation as cellulitis based on the symptoms of redness, pain and swelling may result in unnecessary prescribing of antibiotics as well as delayed referral to lymphoedema services for appropriate management” [5]. The earlier that breast edema is recognized and its underlying source is accurately diagnosed, the more likely physicians are to provide the appropriate targeted intervention on a case-by-case basis. Patients can otherwise succumb to generalized non-targeted remedies and provision of palliative care, which may only provide transitory symptomatic relief rather than actual treatment for the edema.

Toward an objective standardized diagnostic tool

As previously noted, TDC values of breast tissue have emerged as a promising modality to potentially quantify breast edema in a clinically friendly and reliable way [28,53]. TDC values quantify local tissue water content, allowing for measurement of the breast’s baseline tissue water. Ratios obtained from these values can be used to assess breast tissue water content over time and can thus be used to track the status and progression of breast edema. Optimal management of breast edema is contingent on standardizing and utilizing reliable objective diagnostic tools. To this end, it would seem useful to obtain further baseline breast TDC measurements after which such measurements may be used as a component of the standard of care for detecting and monitoring breast edema. Once these parameters are attained and accepted, diagnostic criteria can then be enforced, which, in turn, may propel further research on breast edema to establish treatment guidelines. The utility of TDC measurements for this purpose derives from several factors: measurements are quick, nonintrusive, and accurate, and the device itself is handheld and portable and requires little training for proper use. It is also reasonably cost-effective since there are normally no costs after its initial purchase [48]. Contrastingly, other equipment for such assessment units are often non-portable, significantly more expensive, reliant on fixed landmarks, necessitate training for proper use, time-consuming, and at risk of observer biases [29].

## Conclusions

This comprehensive literature search and critical review on breast edema indicates that evidence-based treatments are not available because no gold standard exists for detecting breast edema. This often leads to physicians neglecting its presence, patients to subsequently suffer various and potentially severe physical and psychological consequences due to their breast edema, and a general lack of understanding among patients and physicians as to how to adequately treat breast edema. This delay in diagnosis enables the disease to progress and symptomatology to worsen, which may lead to inadequate or palliative treatment in the form of chronic antibiotics or pain medications. These measures not only fail to improve the edema but have the potential to exacerbate both the psychological and physical symptoms experienced by patients.

The ability to objectively diagnose breast edema is likely contingent on firstly establishing a proper definition of breast edema that receives an international consensus. Previous studies have attempted to posit a definition, but they have been based on the qualitative aspects of breast edema. Sole reliance on subjective evaluations, which is how breast edema is predominantly assessed by physicians currently, yields inconsistent and sometimes inaccurate detection of the condition, resulting in poor characterization of breast edema overall. What is needed is a fully comprehensive definition inclusive of quantitative aspects of breast edema as well, with research-backed, numerical data that is proven and corroborated by further studies. As such, adequate assessment of the presence or absence of breast edema and measurement of its progression over time can be more easily produced. With improved recognition and awareness of breast edema by physicians, researchers, patients, and patient families, the enigma of breast edema would be unraveled and the quality of life from the physical, psychological, and social standpoints would be considerably improved for the afflicted patient.
